# Protocol for a feasibility randomised controlled trial of the use of Physical ACtivity monitors in an Exercise Referral Setting: the PACERS study

**DOI:** 10.1186/s40814-017-0194-z

**Published:** 2017-12-12

**Authors:** Jemma Hawkins, Michelle Edwards, Joanna Charles, Russell Jago, Mark Kelson, Kelly Morgan, Simon Murphy, Emily Oliver, Sharon Simpson, Rhiannon Tudor Edwards, Graham Moore

**Affiliations:** 1 Centre for the Development and Evaluation of Complex Interventions for Public Health Improvement (DECIPHer), Cardiff University, Cardiff, CF10 3BD UK; 2 Centre for Health Economics and Medicines Evaluation, Bangor University, Bangor, LL57 2PZ UK; 3 Centre for Exercise, Nutrition and Health Sciences, University of Bristol, Bristol, BS8 1TZ UK; 4 Centre for Trials Research, Cardiff University, Cardiff, CF14 4YS UK; 5 School of Applied Social Sciences, Durham University, Durham, DH1 3HN UK; 6 MRC/CSO Social and Public Health Sciences Unit, University of Glasgow, Glasgow, G2 3QB UK

**Keywords:** Exercise referral, Physical activity, Autonomous motivation, Feasibility studies, Accelerometer/try, Physical activity monitors, Physical activity trackers, Costs, Economic evaluation

## Abstract

**Background:**

Exercise referral schemes are recommended by the National Institute for Clinical Excellence (NICE) for physical activity promotion among inactive patients with health conditions or risk factors. Whilst there is evidence for the initial effectiveness and cost-effectiveness of such schemes for increasing physical activity, evidence of long-term effects is limited. Techniques such as goal setting, self-monitoring and personalised feedback may support motivation for physical activity. Technologies such as activity monitoring devices provide an opportunity to enhance delivery of motivational techniques. This paper describes the PACERS study protocol, which aims to assess the feasibility and acceptability of implementing an activity monitor within the existing Welsh National Exercise Referral Scheme (NERS) and proposed evaluation methodology for a full-scale randomised controlled trial.

**Methods/design:**

The PACERS study consists of a pilot randomised controlled trial, process evaluation and exploratory economic analyses. Participants will be recruited from the generic pathway of the Welsh NERS and will be randomly assigned to receive the intervention or usual practice. Usual practice is a 16-week structured exercise programme; the intervention consists of an accelerometry-based activity monitor (MyWellnessKey) and an associated web platform (MyWellnessCloud). The primary outcomes are predefined progression criteria assessing the acceptability and feasibility of the intervention and feasibility of the proposed evaluation methodology. Postal questionnaires will be completed at baseline (time 0: T0), 16 weeks after T0 (T1) and 12 months after T0 (T2). Routinely collected data will also be accessed at the same time points. A sub-sample of intervention participants and exercise referral staff will be interviewed following initiation of intervention delivery and at the end of the study.

**Discussion:**

The PACERS study seeks to assess the feasibility of adding a novel motivational component to an existing effective intervention in order to enhance effects on physical activity and support longer-term maintenance. The study will provide insight into the acceptability of activity-monitoring technologies to an exercise referral population and delivery staff. Data from this study will be used to determine whether and how to proceed to a full-scale trial of effectiveness of the intervention, including any necessary refinements to intervention implementation or the proposed evaluation methodology.

**Trial registration:**

ISRCTN85785652

## Background

Physical inactivity is a major cause of preventable illness with large costs to the National Health Service (NHS) [1]. Increasing activity at the population level and among at-risk groups is a public health priority [2, 3]. Physical activity interventions for at-risk groups often involve advice and/or signposting from primary care practitioners [4]. Exercise referral schemes (ERS) are one common model [5], usually involving referral to a community-based structured exercise programme. In Wales, the 16-week National Exercise Referral Scheme (NERS) has been running since 2007. A previous effectiveness study of the scheme [6] showed that, at 12 months, NERS was associated with improvements in physical activity for patients at risk of coronary heart disease, but not for those referred for anxiety and depression, despite an improvement in their mental health [7]. The evaluation also showed the base-case incremental cost-effectiveness ratio was £12,111 per quality-adjusted life year (QALY), falling to £9741 if participants were to contribute £2 per session [7]. Qualitative data highlighted a need for post-intervention motivational support to maintain changes [7, 8]. Whilst there is evidence for effectiveness of ERS in increasing physical activity in the short term [9–11], evidence of long-term effects is limited. The Department of Health’s Quality Assurance Framework for Exercise Referral [12] highlights the need to understand how to support long-term maintenance of changes in physical activity.

On entry to an ERS, patients may be initially motivated by external sources such as GP advice to attend [13, 14]. However, sustained changes in physical activity are consistently associated with more internalised, or autonomous, motivation [15–17]. According to self-determination theory [18], the development of autonomous motivation can be achieved through supporting psychological needs for autonomy (volitional and self-endorsed engagement), competence (personal mastery and effectiveness) and relatedness (meaningful interpersonal connections). Thus, developing ways to support these three needs should help to maintain changes in physical activity. Support for this notion is provided by the randomised controlled trial of the Welsh NERS which found increases in autonomous motivation after scheme exit. This increase explained almost half of the between-group difference in physical activity 6 months later [19]. Integrating processes to further enhance and sustain autonomous motivation during and after involvement in an exercise referral scheme may lead to larger effects and longer-term maintenance of these. Existing evidence points to potential motivational effects of techniques such as goal setting, monitoring and personalised feedback on progress towards goals [20, 21] which may support autonomous motivation by enhancing patients’ sense of mastery and competence and are recommended by NICE as core components of behaviour change interventions [22].

Technologies such as activity monitors provide opportunities to enhance delivery of goal setting and feedback, allowing for more frequent and automatic feedback on progress towards activity goals, tailored updating of goals based on achievement and remote contact with intervention providers [23]. In addition to addressing psychological needs for competence, incorporation of social components may support motivation through promoting relatedness to other service users. Research on such technologies in exercise interventions suggests that use can be quickly integrated in participants’ lives [24] and may increase physical activity levels [25–29]; however, overall, the evidence is equivocal [23]. Furthermore, little is known about the acceptability of these technologies to ERS populations or if the benefits will remain once the initial novelty has ceased. Exercise referral patients are a diverse group with a range of ages and conditions. For example, although the average age of participants in the evaluation of the Welsh NERS was 52 years old, the overall ages ranged from 16 to 88. Thus, familiarity with technology and willingness to use it may differ within the group [30]. In addition, participant diversity in terms of socioeconomic status and geographic location may result in differences in access to high-speed internet connections or the hardware required for engaging with some technologies (e.g. personal computer). Hence, prior to a trial of effectiveness, which may be undermined by difficulties integrating technologies into routine practice or facilitating uptake by patients, piloting is required to investigate these issues.

A preliminary investigation [31] tested a protocol for integrating activity monitoring devices (MyWellnessKey, Technogym) and a linked web platform in one local authority area of the Welsh NERS. The study showed potential for using the MyWellnessKey (MWK) devices in the scheme; however, further work is required to understand the feasibility and acceptability of this on a larger scale with a demographically diverse population. In this paper, we describe the protocol of the PACERS study, a pilot trial to assess the feasibility and acceptability of using the MWK activity monitors to promote maintenance of physical activity within NERS. The aim of the study is to evaluate the feasibility and acceptability of the intervention (the MWK) and its proposed evaluation methodology, in order to optimise design and delivery and evaluate whether a full-scale randomised controlled trial of effectiveness is warranted and feasible.

### Study aim

The primary aim of the study is to assess the feasibility and acceptability of implementing the MWK activity monitors within the Welsh NERS as well as the proposed evaluation methodology in order to optimise design and delivery for conducting a definitive evaluation trial.

### Study objectives

The main objectives for this study are to investigate:

- the fidelity of delivery of the intervention and trial methodology including compliance with study invitation and randomisation processes;
- the acceptability of the intervention to participants in terms of its usability and likelihood of future use;
- whether randomisation is acceptable to 50% or more of participants;
- the feasibility of recruiting 20% or more new NERS patients and retaining at least 80% of participants at 12-month follow-up (T2);
- contamination, by exploring whether less than 20% of control participants are exposed to the intervention;
- the effect of the intervention on the main hypothesised change mechanism (autonomous motivation); and
- the feasibility of collecting the primary, secondary and process outcome measures and economic evaluation methods.

## Methods

### Study design

**Fig. 1:**
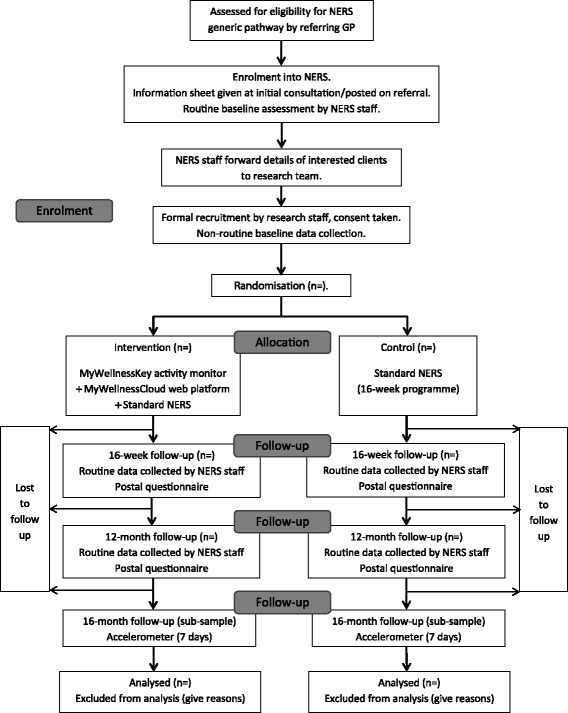
Flow diagram of the PACERS study design

The study design is an individually randomised pilot randomised controlled trial, plus a process evaluation and exploratory economic analyses, of implementing the MWK devices within Welsh NERS standard practice. Data will be collected at three time points: baseline (time 0 (T0)), at the end of the 16-week NERS programme (T1) and 12-months post-baseline (T2). Figure 1 shows the study flow diagram. The study was given favourable ethical opinion for conduct in the NHS on 1 December 2015 by the South East Scotland Research Ethics Committee 02 (REF: 189587).

### Setting and participants

The study is being undertaken within the Welsh NERS across leisure centres in eight local authority areas in Wales, UK. The eight study sites were purposively selected to reflect a range of urbanisation and geography. Participants are eligible for the study if they (i) are referred into the NERS generic pathway (see Table 1) and (ii) have the capacity to use the activity monitors (i.e. computer access and an email address).

**Table 1:** NERS Generic Pathway Criteria

For referral into the NERS generic pathway, patients must:	
- Be aged 16 years or above;	
- Be sedentary (defined as not moderately active for 3 times per week or deconditioned through age or inactivity);	
- Have at least one of the following:	
○ Raised blood pressure 140/90,	
○ BMI > 28,	
○ Cholesterol > 5.0,	
○ Controlled diabetes or impaired glucose intolerance,	
○ Family history of heart disease or diabetes,	
○ At risk of osteoporosis and/or musculoskeletal pain,	
○ Mild arthritis or poor mobility,	
○ Mild-moderate COPD, asthma, bronchitis, emphysema,	
○ Mild anxiety, depression or stress, multiple sclerosis.	

### Recruitment

Participants will be recruited to the trial using opportunistic invites within the existing scheme structure. NERS exercise professionals will provide information about the study to all new generic pathway clients during their first consultation appointment on the scheme. Exercise professionals will transfer the contact details of clients who are eligible for and interested in joining the study to the research team using a secure electronic form. The research team will send a recruitment pack containing full informed consent materials and the baseline questionnaire to interested clients to be returned by post. Participants who return a signed consent form and completed baseline questionnaire will be entered into randomisation. Participants in the intervention group will be sent information about the process evaluation interviews following randomisation and will be asked to express an interest in taking part in the interviews. From those who express an interest, participants will be selected to provide variation in local authority area, age, sex and reason for referral. Where possible, we will interview the same participants at 4 weeks and 12 months. Where not possible, additional participants matched by demographics (e.g. age and sex) will be recruited for 12-month interviews. All NERS staff involved in the study will be invited to participate in the process evaluation interviews. From those who express an interest, two staff members per local authority area will be selected.

### Randomisation

After completion of baseline measures, study staff will randomly assign participants 1:1 to receive either the intervention (NERS plus MWK) or the control treatment (NERS standard practice) via a computer-generated random allocation sequence created by the South East Wales Trials Unit.

### The intervention

The intervention is an enhanced ERS that includes usual care (NERS standard practice) [6] plus an accelorometry-based activity monitor (MyWellnessKey; MWK). The MWK can be used for self-monitoring of physical activity levels in combination with a linked web platform (MyWellnessCloud) and smartphone application (see Table 2). The MWK has been validated in terms of device accuracy at monitoring physical activity level and intensity [32, 33] and utility at fostering increased physical activity levels (high concurrent validity with ActiGraph accelerometer to detect physical activity in laboratory and free-living environments) [34].

**Table 2:** Features of the MWK activity monitor and MyWellnessCloud web platform

• Real-time visual feedback via a screen on the activity monitor	
• Detailed feedback on activity levels via a web platform to indicate progress towards goals, time spent in different activity intensities and calories burned	
• Automatised goal setting via an algorithm which sets goals in a stepwise fashion such that forward progression is mastery-based	
• Facilitation of social support for exercise via the web platform (through involvement in group challenges and remote communication with an exercise professional) and smartphone app (the option to share details about activity completed via social media)	
• Free access to the web platform and smartphone application following discontinuation of use of the MWK via manual input or by linking the account to another monitoring device.	

Intervention participants will be provided with a MWK to use for the remaining 12 weeks of their 16-week NERS programme after receiving it at their 4-week consultation and will be encouraged to use it for 36 weeks after they exit the scheme, up until their 12-month consultation when the device will be returned. In current practice, conducting an 8-month telephone consultation to check clients’ progress with exercise is an optional part of standard care. To encourage participants to maintain engagement in the study, we have asked for the telephone consultation to take place with all intervention participants. Table 3 shows how the intervention will be implemented within the scheme.

**Table 3:** Implementation of the intervention components

Time point	Exercise professionals	Intervention participants
At 4 week review appointment	Set up participants with a MWC account, configure initial activity goals on the MWK and demonstrate how to use the device and web platform.	Take the MWK home, sign into their MWC account on their home computer and connect their MWK to read data and charge it.
Over the study period (48 weeks)	Interact with participants to monitor and adjust their goals, send positive comments and set up group challenges through direct messaging via a linked website called Professional Cloud.	Use the device daily and connect the MWK to a computer at least twice per week to upload data to the MWC, receive feedback and charge the device. Manually enter information about activity that the device does not readily measure, i.e. swimming, weight training, cycling.
At 8 months from start	Telephone participants to check on their progress with exercising and remind them of the study and encourage use of the MWK, MWC and associated features.	Participants with a MWK continue to use it daily.
At 12 months from start	Exercise professionals will have a consultation with all participants for usual NERS assessments and to collect the MWK.	Hand the MWK back to the exercise professional.

**Fig. 2:**
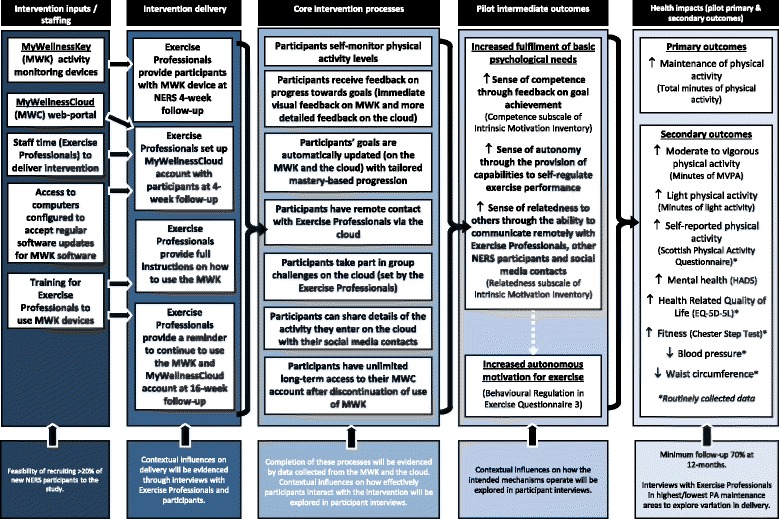
PACERS logic model

It is anticipated that the intervention will enhance NERS through two key mechanisms: (1) goal setting and personalised feedback elements of the devices will support a sense of exercise mastery and perceived competence; (2) the web platform will provide a sense of relatedness to others via opportunities to communicate remotely with exercise professionals, other NERS clients and social media contacts. It is anticipated that these mechanisms will improve autonomous motivation for exercise, leading to greater maintenance of increases in physical activity, as depicted in the intervention logic model (see Fig. 2).

### Control treatment

Control participants will receive usual care which is NERS standard practice: a 16-week structured exercise programme which includes consultations with an exercise professional at the start, 4 weeks, on exiting the scheme (16 weeks) and at 12-month follow-up [6].

### Primary outcome

The primary outcome will be the feasibility and acceptability of the intervention and its proposed evaluation methodology, to inform a decision on whether a full randomised controlled trial is warranted and feasible. This will be assessed against a set of predefined progression criteria related to recruitment and retention rates, exposure to the intervention in both intervention and control groups and acceptability of the intervention, recruitment and randomisation processes to participants. The criteria were agreed by the Trial Steering Committee (TSC) and follow a traffic light assessment system (red = stop; amber = discuss with TSC whether there is enough evidence that sufficient improvements can be made to proceed to full trial without another feasibility assessment; green = proceed) using quantitative measures supported by qualitative data. The criteria, their measurement and assessment criteria are summarised in Table 4. Qualitative data will provide insights into intervention and evaluation design features which need to be retained, or where metrics fall into the amber zone, modifications which may need to be made to improve feasibility and acceptability.

**Table 4:** Summary of progression criteria

Progression criteria (PC)	Measures used	Assessment of whether criteria have been met
PC1. Feasibility to recruit a sufficient proportion of new NERS patients to participate in the trial, with appropriate retention rates to 12-month follow-up.	• The percentage of new NERS patients recruited to the trial, and retained at each subsequent follow-up. • Regression models will be used to identify predictors of loss to follow-up (e.g. demographics or baseline motivation).	• If > 20% of new NERS patients recruited = proceed; if < 5% = full-scale trial unlikely to be feasible. If 5–20% the TSC will consider the feasibility of proceeding to a full-scale trial bearing in mind the data and feedback presented and representativeness of the recruited sample, and possible steps to increase the recruitment rate. • If > 80% retained at 12-months = proceed, if < 60% = full-scale trial unlikely to be feasible. If 60–80% the TSC will consider the feasibility of proceeding based on the available data and possible steps to increase retention.
PC2. Intervention and trial methodology delivered as intended	• Summary statistics for intervention fidelity measures overall and by area. • Compliance with study invite processes. • Compliance with randomisation processes.	• The TSC will consider the data presented and make a judgement about whether the intervention and trial methodology were delivered as intended.
PC3. At least one of the two intervention components is acceptable to participants	• Percentages of participants who report acceptability of the intervention components on four self-report questions. • Issues regarding acceptability of, and engagement with, the two intervention components explored in qualitative interviews with a sub-sample of intervention participants.	• The TSC will consider the quantitative and qualitative data and make an overall judgement on whether the intervention is acceptable.
PC4. Recruitment and randomisation processes acceptable to > 50% of recruited participants	• Percentages of participants who report acceptability of the recruitment and randomisation processes on patient questionnaires. • Exploration of understanding and acceptability of recruitment and randomisation processes in qualitative interviews.	• > 50% of recruited participants report ‘agree’ or ‘strongly agree’ to questions about the acceptability of recruitment and randomisation processes. • The TSC will apply discretion in judging whether this criterion has been met, or could be addressed to improve acceptability in a full-scale trial.
PC5. < 20% of control group exposed to the intervention components	• The percentage of participants in intervention and control groups who report that they were provided with a MWK device or accessed the MWC web platform.	• < 20% of control participants report that they have used a MWK device during the study period. • < 20% of control participants report that they have accessed the MWC during the study period.

It is anticipated that in a full trial, the main outcome measure will be objectively measured physical activity using accelerometry. To examine the feasibility of collecting this data at follow-up in the NERS population, a sub-sample of participants will be recruited to complete the accelerometer measure at 16 months post-randomisation. Participants will wear a GT3X ActiGraph accelerometer around the waist for seven consecutive days during waking hours. Data will be processed to identify mean minutes of moderate to vigorous intensity activity per day and mean accelerometer counts per minute (volume of physical activity) using established processes [35].

### Secondary outcomes

The effect of the intervention on the main hypothesised change mechanism (autonomous motivation) will be evaluated. Other secondary outcome measures will be piloted to estimate key trial parameters (e.g. standard deviation) to inform a future full trial.

#### Measures collected routinely in NERS

Data collected routinely within NERS will be obtained for use within the trial from T0, T1 and T2, as follows:

- Blood pressure and resting heart rate;
- Body mass index;
- Waist circumference;
- Self-reported physical activity (Scottish Physical Activity Questionnaire) [36];
- Health-related quality of life (EQ-5D-5L) [37]; and
- Fitness test (Chester fitness test) [38].

#### Measures included in PACERS study questionnaire

The following additional measures will be collected at all time points, which in a full trial would be used to assess effectiveness of the added intervention component (MyWellnessKey):

- Autonomous Motivation (Behavioural Regulation in Exercise Questionnaire 3 (BREQ-3)) [39];
- Psychological need support (Intrinsic Motivation Inventory) [40];
- Anxiety and depression (Hospital Anxiety and Depression Scale (HADS)) [41].

#### Economic evaluation outcome measures

The PACERS study questionnaire will include an adapted Client Service Receipt Inventory (CSRI) based on the previous service use questionnaire used in the NERS evaluation [7] and examples in the DIRUM database (dirum.org) to capture client health and social care service use since the last time point (plus a 4 month retrospective period at baseline). Additional questions in the 12-month questionnaire will capture wider economic outcomes including current work status, days off work due to health problems and estimated income lost due to changes in work during the study period. Willingness to pay for the MWK will also be explored. Baseline demographic data on housing status and household income will also be collected in the PACERS study questionnaire for the purpose of the economic analysis.

### Sample size

The proposed sample size for the study of 286 participants was calculated to allow the estimation of the feasibility proportions of adherence and retention to within at least plus or minus 8.2 percentage points using a 95% confidence interval, as well as to provide 80% power to detect an effect size of 0.4 at the 5% level on the main hypothesised mediator of autonomous motivation at 12-month follow-up, assuming 30% attrition [7]. The sample size was also planned to provide an indication of likely response rates, permit estimates of effect sizes of primary and secondary outcomes in advance of a larger trial and allow exploration of socio-demographic patterning in uptake and use of the MWKs in order to generate hypotheses regarding who the intervention might work for and why.

### Data collection

**Fig. 3:**
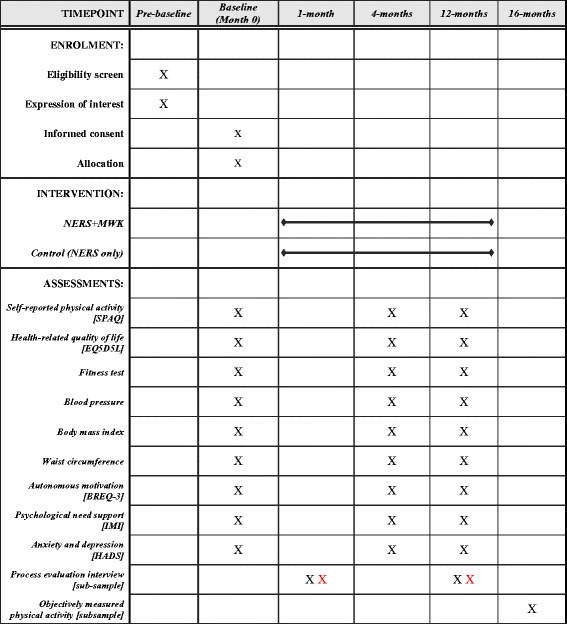
PACERS study schedule of enrolment, interventions and assessments. *Black-coloured* X indicates study participants and *red-coloured* X indicates intervention delivery staff

Routinely collected data will be extracted from the NERS database at all T0, T1 and T2. The PACERS study questionnaire will be mailed to participants at all time points. Telephone and email reminders will be made to non-responders. Semi-structured telephone interviews will be conducted with a sub-sample of intervention participants (*n* = 20) following receipt of the intervention at 4 weeks and again at 12 months (T2) to explore feasibility and acceptability of the intervention and study methods. In addition, telephone interviews will be conducted with a sample of NERS exercise professionals at the same time points to explore feasibility and acceptability of implementing the intervention and study methods from a professional perspective. Figure 3 indicates the schedule of enrolment, interventions and assessments.

### Process evaluation

A detailed process evaluation will examine the acceptability and feasibility of the intervention and evaluation methods, including intervention delivery and fidelity, potential contamination and contextual influences. Quantitative and qualitative data will be collected using a range of methods. Table 5 summarises the process evaluation methods.

**Table 5:** Summary of process evaluation methods

Fidelity/feasibility/acceptability	Method of data collection	Aims to explore	Method of analysis/data to be presented	Participants	Time
Fidelity to trial methodology (PC2)	Audio recordings of NERS initial consultations with participants	The accuracy with which recruitment and consent processes were followed.	A summary score of adherence to the processes (range 0–4) will be calculated for each recording and presented overall and by area.	Two participants per exercise professional	T0 (during NERS initial consultation)
Feasibility of implementing the intervention and trial methodology within routine NERS practice	Telephone interviews with NERS staff	Barriers/facilitators, fit with local context, any adverse effects on usual NERS delivery, differences across settings, additional infrastructure or resources required for a full trial.	Thematic analysis.	Two exercise professionals per area	After receipt of the intervention at 4 weeks and at T2
Acceptability of the trial methodology (PC4)	Telephone interviews with NERS staff and intervention participants	Understandings and acceptability of recruitment and randomisation processes.	Thematic analysis.	Two exercise professionals per area, 20 intervention participants	After receipt of the intervention at 4 weeks and at T2
	Self-report questions on study questionnaire		Percentages of participants reporting acceptability of the randomisation process.	All participants	T1
Acceptability of the intervention (PC3)	Telephone interviews with professionals and participants patients	Perceived acceptability of intervention components, barriers and facilitators in using the devices.	Thematic analysis.	Two exercise professionals per area, 20 intervention participants	After receipt of the intervention at 4 weeks and at T2
	Self-report questions on study questionnaire	Frequency of use, ease of use, likelihood of future use.	Percentages of participants reporting that the intervention was easy to use, that they used it, and would do so in the future.	All intervention participants	T1 and T2
Feasibility of collecting objective data on physical activity at long-term follow-up	ActiGraph accelerometers	The feasibility of obtaining measures of physical activity over a 7 day period.	A linear regression model controlling for age, gender, baseline self-reported physical activity and randomisation group will be fitted. Results will be expressed using regression coefficients, 95% confidence intervals, and standardised effect sizes.	100 participants	16 months post-randomisation
Contamination (PC5)	Self-report questions on study questionnaire on awareness of and exposure to intervention components	Assessment of contamination between trial arms.	Percentages of participants in intervention and control arms reporting exposure to the intervention will be presented alongside 95% confidence intervals.	All participants	T1 and T2

### Economic analysis methods

Data will be collected to estimate intervention costs and examine the feasibility of calculating cost-effectiveness alongside a definitive full pragmatic randomised trial. Health care service use will be costed using national unit costs [42, 43]. Both arms of the study with be costed, revisiting and revising the costing methodology used in previous economic analysis of the Welsh NERS [44].

The additional costs of the intervention will consist of the cost of the MWK; staff costs relating to the MWK (e.g. training, implementation and participant follow-up support); the cost of the professional web cloud (e.g. licence fee) and additional staff interactions. These costs are in addition to the core programme costs (in both arms) including NERS standard practice costs and participant contributions. Information about the additional staff resources required for the use of the MWK and professional web cloud will be derived from qualitative interviews with staff.

### Data analysis

#### Quantitative analysis

The main outcomes in this feasibility study are related to the study progression criteria as outlined in Table 4. The methods of analysis for quantitative data collected for the process evaluation are summarised in Table 5. Analyses will be largely descriptive, with summary statistics being presented overall and also by key demographics. Evidence of whether the intervention could lead to behaviour change will be examined using regression analyses to quantify effects on autonomous motivation, using the Relative Autonomy Index derived from the BREQ-3.

To examine the direction of effect on physical activity, analysis of covariance models [ANCOVA] will be used to estimate intervention effects on physical activity at 16 months. Whilst likely non-significant due to limited power, this should be in the direction of a favourable intervention effect. Accelerometer data will be processed using standard procedures; periods of ≥ 60 min of zero counts will be recorded as “non-wear time” and removed. Participants will be included in the analysis if they provide ≥ 3 valid days (i.e. 500 min of data between 6 am and 11 pm). Mean minutes of daily moderate to vigorous intensity activity will be estimated using a threshold value of ≥ 2020 counts per minute with minutes of light intensity physical activity estimated using thresholds of between 100 and 2019 counts per minute [35]. Sedentary time will be estimated based on a cut-point of less than 100 counts per minute; mean sedentary minutes per day will be derived.

#### Qualitative analysis

Qualitative data from interviews with exercise professionals and intervention participants will be transcribed verbatim and organised and coded into a thematic framework using NVivo 11 software. An approach to thematic analysis will be used that allows for both a deductive and inductive approach to data analysis [45]. Data will be initially coded using an a priori coding scheme of categories which align with the research questions as a means of organising the data for subsequent interpretation. An element of flexibility will be maintained to account for the emergence of any new and unexpected themes. The first three transcripts will be independently coded by two coders in order to develop a shared codebook via consensus. Any disagreements between coders will be discussed with a third coder. Divergence and convergence between interviews will be examined and comparisons made of the experiences of the intervention across and within areas (NERS clients and exercise professionals). We aim to develop a comprehensive understanding of the intervention acceptability, implementation and mechanisms of impact.

#### Economic analysis

A pilot cost-consequence analysis will be conducted from a NHS and societal perspective. Response rates and level of completion of the measures will be reported using descriptive statistics. Variables will be checked for out of range values before analysis begins. As data are expected to be skewed, non-parametric tests will be used to assess differences across groups or time points for the outcomes of QALYs (using the EQ-5D) and health and social care service use. We will bootstrap (5000 replications) differences in cost and outcomes to produce a 95% confidence interval around these differences. Ceiling effects on the EQ-5D will also be assessed, assessing the proportion of participants that state “no problems” on all five dimensions on the EQ-5D questionnaire. QALY gains (using the EQ-5D) will be compared to those in similar samples from previous literature (where available).

A report on the data gathered about service use (from routinely collected data recorded by healthcare professionals delivering NERS) will explore if future studies could use this or a different method to the CSRI questionnaire used in the feasibility study. Descriptive statistics will be used to describe the amount participants are willing to pay for the MWK, both during the intervention and beyond. Response rates and level of completion of the questions exploring how best to capture productivity losses will be reported using descriptive statistics.

Sub-group analyses will explore the effect on health-related quality of life of socio-demographics (e.g. gender) and reason for referral. Sensitivity analysis will be conducted in accordance with NICE guidelines to vary the cost of the device [46], demonstrating what happened in the feasibility trial and how co-ordination may be varied in a future full-scale trial.

### Serious adverse event reporting and monitoring

It is not anticipated that there will be any additional risks to participants over and above existing NERS standard practice for which standard operating procedures are in place covering referral into the scheme, provision of exercise instruction and support, and dealing with adverse events. There are no serious adverse events expected to be related to the intervention. Any serious adverse event occurrence will be reported to the Chief Investigator within 48 h of receiving notification. Assignment of causality will be made by the independent clinician member of the TSC.

### Project management

A Trial Management Group (TMG) is responsible for ensuring the appropriate, effective and timely implementation of the trial including monitoring adherence to standardised research protocols. The day-to-day operational management of the feasibility study is co-ordinated by a central project management team which meets weekly to monitor progress and any issues which may need relaying to the TMG. An independent TSC provides overall supervision for the trial and advice through its independent chair and also encompasses the role of Data Monitoring Committee.

## Discussion

The PACERS feasibility trial aims to assess the feasibility and acceptability of implementing a novel motivational component, the MyWellnessKey, into the existing Welsh NERS. In addition, the trial also aims to determine the acceptability and feasibility of the proposed evaluation methodology for a definitive trial of the intervention for promoting long-term maintenance of physical activity. Whilst exercise referral approaches have been shown to be effective for increasing physical activity levels, evidence of long-term effects is limited [9, 10, 12] and so there is a need to better understand how to support long-term maintenance of physical activity [3]. The MWK intervention offers a potential mechanism for enhancing and sustaining autonomous motivation for physical activity via evidence-based techniques including goal setting, self-monitoring and receiving personalised feedback on progress towards goals [20–22].

Findings from this study will determine whether progression to a full-scale randomised controlled trial of effectiveness and cost-effectiveness is feasible and warranted, through the assessment of key progression criteria. The study will assess whether the outcomes being used are feasible and acceptable to use with the study population. Findings related to the acceptability and feasibility of implementing the intervention will inform potential refinement of the implementation processes where necessary. The findings will also allow refinement of the intervention logic model.

## Abbreviations

CSRI: Client service receipt inventory
ERS: Exercise referral scheme
MWC: MyWellnessCloud
MWK: MyWellnessKey
NERS: National Exercise Referral Scheme
NHS: National Health Service
NICE: National Institute for Clinical Excellence
NRES: National Research Ethics Service
QALY: Quality-adjusted life year
REC: Research Ethics Committee
TSC: Trial Steering Committee

## References

[1] British Heart Foundation. The economic costs of physical inactivity. British Heart Foundation National Centre. Loughborough: Loughborough University: 2013.

[2] Department of Health (2011). Start active, stay active: a report on physical activity from the four home countries.

[3] NICE (2014). Exercise referral schemes to promote physical activity.

[4] Din NU, Moore G, Murphy S, Wilkinson C, Williams NH (2014). Health professionals’ perspectives on exercise referral and physical activity promotion in primary care: findings from a process evaluation of the National Exercise Referral Scheme in Wales. Health Educ J.

[5] Sowden SL, Raine R (2008). Running along parallel lines: how political reality impedes the evaluation of public health interventions. A case study of exercise referral schemes in England. J Epidemiol Community Health.

[6] Murphy S, Raisanen L, Moore G, Edwards RT, Linck P, Williams N (2010). A pragmatic randomised controlled trial of the Welsh National Exercise Referral Scheme: protocol for trial and integrated economic and process evaluation. BMC Public Health.

[7] Murphy SM, Edwards RT, Williams N, Raisanen L, Moore G, Linck P (2012). An evaluation of the effectiveness and cost effectiveness of the National Exercise Referral Scheme in Wales, UK: a randomised controlled trial of a public health policy initiative. J Epidemiol Community Health.

[8] Moore GF, Raisanen L, Moore L, Ud Din N, Murphy SM (2013). Mixed-method process evaluation of the Welsh National Exercise Referral Scheme. Health Educ J.

[9] Pavey TG, Taylor AH, Fox KR, Hillsdon M, Anokye N, Campbell JL, et al. Effect of exercise referral schemes in primary care on physical activity and improving health outcomes: systematic review and meta-analysis. BMJ. 2011;343:d6462. 10.1136/bmj.d6462 PMC3209555 22058134

[10] Campbell F, Holmes M, Everson-Hock E, Davis S, Buckley Woods H, Anokye N (2015). A systematic review and economic evaluation of exercise referral schemes in primary care: a short report. Health Technol Assess.

[11] Williams NH, Hendry M, France B, Lewis R, Wilkinson C (2007). Effectiveness of exercise-referral schemes to promote physical activity in adults: systematic review. Br J Gen Pract.

[12] Department of Health (2001). Exercise referral systems: a national quality assurance framework.

[13] Rouse PC, Ntoumanis N, Duda JL, Jolly K, Williams GC (2011). In the beginning: role of autonomy support on the motivation, mental health and intentions of participants entering an exercise referral scheme. Psychol Health.

[14] Markland D, Tobin VJ (2010). Need support and behavioural regulations for exercise among exercise referral scheme clients: the mediating role of psychological need satisfaction. Psychol Sport Exerc.

[15] Teixeira PJ, Silva MN, Mata J, Palmeira AL, Markland D (2012). Motivation, self-determination, and long-term weight control. Int J Behav Nutr Phys Act.

[16] Teixeira PJ, Carraça EV, Markland D, Silva MN, Ryan RM (2012). Exercise, physical activity, and self-determination theory: a systematic review. Int J Behav Nutr Phys Act.

[17] Fortier MS, Wiseman E, Sweet SN, O'Sullivan TL, Blanchard CM, Sigal RJ (2011). A moderated mediation of motivation on physical activity in the context of the PAC randomized control trial. Psychol Sport Exerc.

[18] Deci EL, Ryan RM (2000). The ‘what’ and ‘why’ of goal pursuits: human needs and the self-determination of behavior. Psychol Inq.

[19] Littlecott HJ, Moore GF, Moore L, Murphy S (2014). Psychosocial mediators of change in physical activity in the Welsh national exercise referral scheme: secondary analysis of a randomised controlled trial. Int J Behav Nutr Phys Act.

[20] O'Brien N, McDonald S, Araujo-Soares V, Lara J, Errington L, Godfrey A (2015). The features of interventions associated with long-term effectiveness of physical activity interventions in adults aged 55-70 years: a systematic review and meta-analysis. Health Psychol Rev.

[21] Shilts MK, Horowitz M, Townsend MS (2004). Goal setting as a strategy for dietary and physical activity behavior change: a review of the literature. Am J Health Promot.

[22] NICE (2014). Behaviour change: individual approaches.

[23] Petit A, Cambon L (2016). Exploratory study of the implications of research on the use of smart connected devices for prevention: a scoping review. BMC Public Health.

[24] Hunt K, McCann C, Gray CM, Mutrie N, Wyke S (2013). “You’ve got to walk before you run”: positive evaluations of a walking program as part of a gender-sensitized, weight-management program delivered to men through professional football clubs. Health Psychol.

[25] Cupples M, Dean A, Tully MA, Taggart M, McCorkell G (2013). Using pedometer step-count goals to promote physical activity in cardiac rehabilitation: a feasibility study of a controlled trial. Int J Phys Med Rehabil.

[26] Vaes AW, Cheung A, Atakhorrami M, Groenen MT, Amft O, Franssen FM (2013). Effect of ‘activity monitor-based’ counseling on physical activity and health-related outcomes in patients with chronic diseases: a systematic review and meta-analysis. Ann Med.

[27] Davies CA, Spence JC, Vandelanotte C, Caperchione CM, Mummery WK (2012). Meta-analysis of internet-delivered interventions to increase physical activity levels. Int J Behav Nutr Phys Act.

[28] Kirwan M, Duncan JM, Vandelanotte C, Mummery KW (2012). Using smartphone technology to monitor physical activity in the 10,000 steps program: a matched case control trial. J Med Internet Res.

[29] Butryn ML, Arigo D, Raggio GA, Colasanti M, Forman EM (2014). Enhancing physical activity promotion in midlife women with technology-based self-monitoring and social connectivity: a pilot study. J Health Psychol.

[30] Olson KE, O’Brien MA, Rogers WA, Charness N (2011). Diffusion of technology: frequency of use for younger and older adults. Ageing international.

[31] Hawkins JL, Oliver EJ, Wyatt-Williams J, Scale E, van Woerden HC (2014). Assessing the feasibility of using uniaxial accelerometers with an online support platform in the delivery of a community-based exercise referral scheme. J Prim Care Community Health.

[32] Bergamin M, Ermolao A, Sieverdes JC, Zaccaria M, Zanuso S (2012). Validation of the mywellness key in walking and running speeds. J Sports Sci Med.

[33] Sieverdes JC, Wickel EE, Hand GA, Bergamin M, Moran RR, Blair SN (2013). Reliability and validity of the Mywellness Key physical activity monitor. Clin Epidemiol.

[34] Herrmann SD, Hart TL, Lee CD, Ainsworth BE (2011). Evaluation of the MyWellness Key accelerometer. Br J Sports Med.

[35] Troiano RP, Berrigan D, Dodd KW, Masse LC, Tilert T, McDowell M (2008). Physical activity in the United States measured by accelerometer. Med Sci Sports Exerc.

[36] Lowther M, Mutrie N, Loughlan C, McFarlane C (1999). Development of a Scottish physical activity questionnaire: a tool for use in physical activity interventions. Br J Sports Med.

[37] The EuroQol Group. EuroQol—a new facility for the measurement of health-related quality of life. Health Policy. 1990;16(3):199–208. 10.1016/0168-8510(90)90421-9 10109801

[38] Sykes K (1998). Chester step test; resource pack (version 3).

[39] Markland D, Tobin V (2004). A modification of the behavioral regulation in exercise questionnaire to include an assessment of amotivation. J Sport Exerc Psychol.

[40] McAuley E, Duncan T, Tammen VV (1989). Psychometric properties of the intrinsic motivation inventory in a competitive sport setting: a confirmatory factor analysis. Res Q Exerc Sport.

[41] Zigmond AS, Snaith RP (1983). The hospital anxiety and depression scale. Acta Psychiatr Scand.

[42] Curtis L, Burns A. Unit Costs of Health and Social Care. Canterbury: Personal Social Services Research Unit; 2016.

[43] Department of Health (2016). NHS reference costs.

[44] Edwards RT, Linck P, Hounsome N, Raisanen L, Williams N, Moore L (2013). Cost-effectiveness of a national exercise referral programme for primary care patients in Wales: results of a randomised controlled trial. BMC Public Health.

[45] Fereday J, Muir-Cochrane E (2006). Demonstrating rigor using thematic analysis: a hybrid approach of inductive and deductive coding and theme development. Int J Qual Methods.

[46] Andronis L, Barton P, Bryan S (2009). Sensitivity analysis in economic evaluation: an audit of NICE current practice and a review of its use and value in decision-making. Health Technol Assess.

